# Obstacle Avoidance of Two-Wheel Differential Robots Considering the Uncertainty of Robot Motion on the Basis of Encoder Odometry Information

**DOI:** 10.3390/s19020289

**Published:** 2019-01-12

**Authors:** Jiyong Jin, Woojin Chung

**Affiliations:** School of Mechanical Engineering, Korea University, Seoul 02841, Korea; jin8644@korea.ac.kr

**Keywords:** mobile robot, motion uncertainty, wheel encoder, path planning

## Abstract

It is important to overcome different types of uncertainties for the safe and reliable navigation of mobile robots. Uncertainty sources can be categorized into recognition, motion, and environmental sources. Although several challenges of recognition uncertainty have been addressed, little attention has been paid to motion uncertainty. This study shows how the uncertainties of robot motions can be quantitatively modeled through experiments. Although the practical motion uncertainties are affected by various factors, this research focuses on the velocity control performance of wheels obtained by encoder sensors. Experimental results show that the velocity control errors of practical robots are not negligible. This paper proposes a new motion control scheme toward reliable obstacle avoidance by reflecting the experimental motion uncertainties. The presented experimental results clearly show that the consideration of the motion uncertainty is essential for successful collision avoidance. The presented simulation results show that a robot cannot move through narrow passages owing to a risk of collision when the uncertainty of motion is high. This research shows that the proposed method accurately reflects the motion uncertainty and balances the collision safety with the navigation efficiency of the robot.

## 1. Introduction

It is important to overcome different types of uncertainties for the safe and reliable navigation of mobile robots. Three main categories of uncertainties can be identified: uncertainties in recognition, motion, and the environment [1,2,3,4]. Recognition uncertainties are caused by the practical limitations of sensors or algorithms; for instance, the position uncertainty of obstacles caused by the sensor error. Environmental uncertainties arise from the inaccurate representation or dynamic change of the environment. Changes in the environment, such as large parking spaces or exhibition halls with dynamic obstacles, can cause the localization to become uncertain [5]. Sources of motion uncertainties include controller errors, latency, disturbances, and modeling errors [6]. Uncertainties may arise when robotically-steering flexible medical needles to clinical targets in soft tissues [7] and motion control of service robots passing between narrow and long obstacles. Thus far, several challenges in recognition and environmental uncertainties have been addressed. Little attention has been paid to motion uncertainties.

Several studies have focused on collision avoidance problems. Fox proposed a dynamic window approach (DWA); the DWA is widely used owing to its simplicity and smooth motions in a dynamic environment [8]. Brock extended the conventional DWA to a global dynamic window approach (GDWA) in order to guide robots in complex environments [9]. Minguez proposed a nearness diagram (ND) method, where robots exhibited great collision avoidance performances in cluttered environments [10]. Borenstein proposed a vector field histogram (VFH) method that enabled robust performances with respect to sensor errors [11]. Zi proposed a collision avoidance method for an omni-directional mobile robot in Ref. [12] and a collision avoidance method for multiple parallel mobile cranes (CPRMCs) in Ref. [13]. Various conventional collision avoidance algorithms are still widely used in applications.

Control strategies toward safe navigation have been extensively studied. In the authors’ prior work [14], collisions with dynamic obstacles from occluded regions were considered. It was shown that the limitations of visibility can be overcome by appropriate path planning and speed control strategies. Roy proposed an intelligent navigation scheme that can improve performance by learning the collision probability [15]. A sampling-based planner has been developed that achieves safety by maximizing the margin to obstacles in the input space [16]. A speed control strategy under the consideration of map and motion uncertainties has also been proposed [17].

Some obstacle avoidance schemes quantitatively consider the risk of collision. So far, various indices have been developed for quantitative collision risk evaluation. Kuffner proposed a region of inevitable collision (RIC) scheme [18]. The RIC extends the obstacle region with respect to robot motion. Frichard proposed an inevitable collision area for a mobile robot [19]. Zucker introduced a relative collision risk [20]. Chung proposed the collision risk index (CRI) that represents the margin of velocity control in the input space [14]. Horst proposed a method to define an appropriate time-to-collision (TTC) [21]. ISO 17387 specifies the TTC level for a collision warning function in a vehicle-mounted crash avoidance system (CAS) [22].

The defining point of this study is that it is important to model motion uncertainties of practical robots quantitatively. Motion uncertainties may vary with the type of robot used. Although the accurate control of the actuator velocity is not a difficult problem in recent days, many commercially-available robots show unsatisfactory velocity control performances. Many research studies recommend the intentional inflation of uncertainty in order to consider various unknown uncertainties [1,16,17,23]. Previous research included the extension of the obstacle area to reduce the risk of collision. However, it is useless for the performance and usability of the robot if the robot is moved by excessively expanding obstacles.

It is clear that the inflation should start from the estimated uncertainty of the given robots. After accurately modeling the uncertainty, the controller finally perceives the degree by which the obstacle has enlarged. Therefore, it is better to model the uncertainty of the robot accurately and to expand the obstacle area to the size of the model. The key ideas of this paper are that the modeling and exploitation of the motion uncertainties are extremely significant for practical applications. The aim of this paper is to achieve the safe and efficient navigation of mobile robots under the consideration of the motion uncertainties.

This study shows how the uncertainty of robot motions is experimentally modeled. The motion uncertainty is assumed to be represented by the velocity control error of a wheel. In other words, the velocity control error is assumed to be a dominant source of motion uncertainty. Then, the modeled uncertainty is reflected in the design of a motion control scheme. The proposed scheme is verified through simulations and experiments. The presented results clearly indicate that the resulting movements of a robot exhibit significant differences under different uncertainty conditions that are considered. It can be concluded that it is essential to model and exploit motion uncertainties.

## 2. Experimental Modeling of the Motion Uncertainty Using Encoder Odometry Information

**Figure 1:**
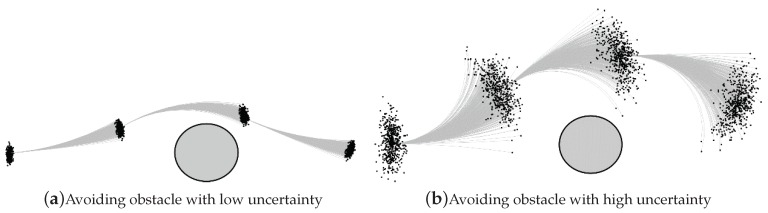
Illustration of the motion uncertainties during obstacle avoidance.

This section describes how to model the uncertainty of robot motions. It is difficult to obtain conventional collision avoidance algorithms that explicitly consider the motion uncertainty of a robot. The uncertainty of the robot position increases with increasing velocity [17]. The motion uncertainty should be modeled with the consideration of the type of robot and motion control performance. Sources of motion uncertainty include unmodeled latency, inaccurate parameters, and disturbances. In this study, the velocity control error obtained by an encoder sensor is assumed to be a dominant source of motion uncertainty. Figure 1 illustrates the effect of motion uncertainties when a robot moves around an obstacle. If the motion uncertainty is low, as shown in Figure 1a, the vehicle travels close to the obstacle. As shown in Figure 1b, if the uncertainty is high, the robot travels away from the obstacles. It may be safe to navigate at distances sufficiently far from the obstacles. However, maintaining an excessive distance from an obstacle reduces the robot’s traveling efficiency. Therefore, accurate modeling of the motion uncertainty is needed to maintain both the navigation efficiency and safety.

The translational and rotational velocities of a two-wheeled mobile robot can be given as follows:

(1) $$v=\frac{v_{l}+v_{r}}{2}$$

(2) $$\omega =\frac{v_{r}+v_{l}}{b}$$

The translational velocity is denoted by *v*, and the rotational velocity is denoted by ω. The tread *b* is assumed to be constant. $v_{l}$ and $v_{r}$ represent the velocity of the left and right wheels, respectively. The velocity control error of both wheels represents the motion uncertainty of a two-wheeled differential robot. The velocity error can be obtained from the difference between the reference and experimental velocities. Thus, the motion uncertainty of a robot can be obtained through practical experiments.

## 3. Motion Controller Considering the Uncertainty of Robot Motion

It was assumed that the velocity error follows a Gaussian distribution. For a reference velocity $x=[v_{l\_ref};v_{r\_ref}]$, experimental velocity mean error $M=[v_{l\_exp};v_{r\_exp}]$, and velocity error covariance matrix $\Sigma =[\begin{array}{cc} \sigma_{l} & 0 \end{array};\begin{array}{cc} \sigma_{r} & 0 \end{array}]$, the multivariate Gaussian probability can be given by Equation (3) [24]. $\sigma_{l}$ and $\sigma_{r}$ denote the standard deviations in velocity errors of the left and right wheels obtained through encoders, respectively.

(3) $$\begin{array}{cc} p(x|M,\Sigma ) & =\frac{1}{2\pi \sqrt{\left|\begin{array}{c} \sigma_{l}^{2}\;0\\ 0\;\sigma_{r}^{2} \end{array}\right|}}\exp\left\{-\frac{1}{2}{\left[\begin{array}{c} v_{l\_err}\\ v_{r\_err} \end{array}\right]}^{T}{\left[\begin{array}{c} \sigma_{l}^{2}\;0\\ 0\;\sigma_{r}^{2} \end{array}\right]}^{-1}\left[\begin{array}{c} v_{l\_err}\\ v_{r\_err} \end{array}\right]\right\}\\ & =\frac{1}{2\pi \sigma_{l}\sigma_{r}}\exp\left\{-\frac{1}{2}{\left[\begin{array}{c} v_{l\_err}\\ v_{r\_err} \end{array}\right]}^{T}\left[\begin{array}{c} \frac{1}{\sigma_{l}^{2}}\;0\\ 0\;\frac{1}{\sigma_{r}^{2}} \end{array}\right]\left[\begin{array}{c} v_{l\_err}\\ v_{r\_err} \end{array}\right]\right\} \end{array}$$

In Equation (3), $v_{l\_err}≜v_{l\_ref}-v_{l\_exp}$ and $v_{r\_err}≜v_{r\_ref}-v_{r\_exp}$ are defined. Since the velocities of both wheels of a two-wheeled mobile robot are independent, the covariance matrix is diagonal. The inverse of *s* can be simply calculated by taking the reciprocal of each diagonal element. Equation (3) is expanded as follows:

(4) $$\begin{array}{cc} p(x|M,\Sigma ) & =\frac{1}{2\pi \sigma_{l}\sigma_{r}}\exp\left\{-\frac{1}{2}{\left[\begin{array}{c} v_{l\_err}\\ v_{r\_err} \end{array}\right]}^{T}\left[\begin{array}{c} \frac{1}{\sigma_{l}^{2}}v_{l\_err}\\ \frac{1}{\sigma_{r}^{2}}v_{r\_err} \end{array}\right]\right\}\\ & =\frac{1}{2\pi \sigma_{l}\sigma_{r}}\exp\left\{-\frac{1}{2\sigma_{l}^{2}}{\left(v_{l\_err}\right)}^{2}-\frac{1}{2\sigma_{r}^{2}}{\left(v_{r\_err}\right)}^{2}\right\}\\ & =\frac{1}{\sqrt{2\pi }\sigma_{l}}\exp\left\{-\frac{1}{2\sigma_{l}^{2}}{\left(v_{l\_err}\right)}^{2}\right\}\cdot \frac{1}{\sqrt{2\pi }\sigma_{r}}\exp\left\{-\frac{1}{2\sigma_{r}^{2}}{\left(v_{r\_err}\right)}^{2}\right\} \end{array}$$

**Figure 2:**
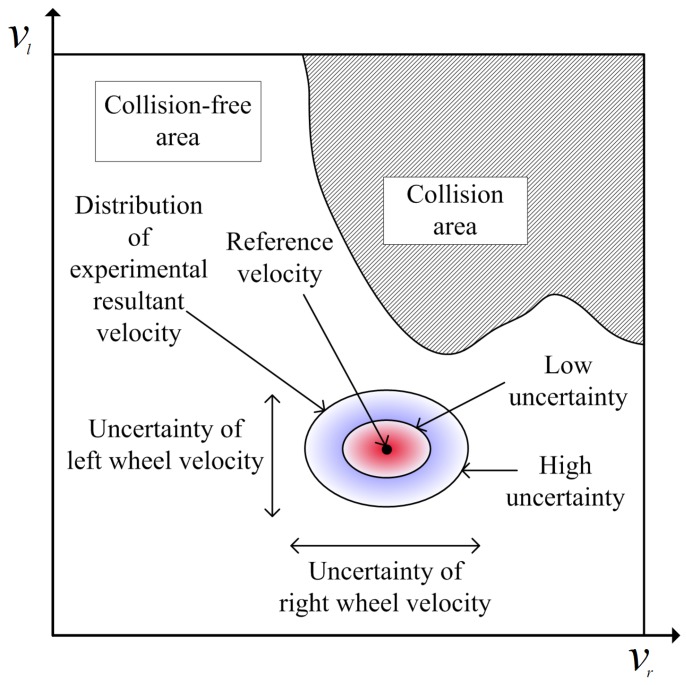
Uncertainty ellipse around the reference velocity in the input space.

Here, the last equation is the product of two independent Gaussian distributions. If the input space consists of $v_{l}$ and $v_{r}$ and the velocity error distribution is represented by a covariance ellipse, the semi-major and semi-minor axes are parallel to the $v_{l}$ and $v_{r}$ axes, respectively. The velocity uncertainties of $v_{l\_ref}$ and $v_{r\_ref}$ in the input space can be expressed by an elliptic inequality. This elliptic inequality can be obtained by the confidence coefficient, covariance matrix, and velocity control error.

(5) $$\frac{{\left(v_{l\_err}\right)}^{2}}{\sigma_{l}^{2}}+\frac{{\left(v_{r\_err}\right)}^{2}}{\sigma_{r}^{2}}\leq s$$

where *s* is the critical value of the chi-squared distribution. Figure 2 shows the uncertainty ellipse, which is the expansion of the input velocity under the consideration of velocity control errors. If a robot with a large motion uncertainty chooses a velocity near the obstacle area, the robot is more likely to collide with obstacles. Therefore, knowledge of the magnitude of the uncertainty associated with the current traveling condition of the robot is required. If the size is known through experiment, a safe speed can be selected by expanding the obstacle by the amount of motion uncertainty. The shape of the motion uncertainty ellipse depends on the magnitude of the velocity uncertainty of the left and right wheels. If the motion of the robot is accurate, the uncertainty ellipse is small. However, the risk of collision increases according to increases in the motion uncertainty.

**Figure 3:**
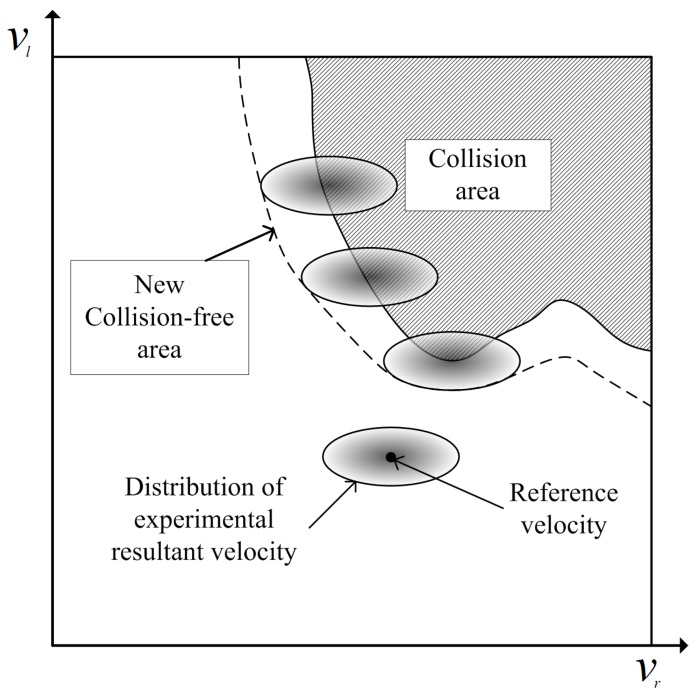
Uncertainty ellipse around the reference velocity in the input space.

Therefore, the clearance considering the uncertainty of the robot motion (CURM) is proposed as shown in Figure 3. Using the CURM, experimental resultant velocities of a wheel remain inside the collision-free input region, regardless of the uncertainty. The CURM means the expected value of the clearance considering the motion uncertainty. The CURM is the smallest value in the uncertainty ellipse of the reference velocity. The extent of the obstacle expansion depends on the type of robot and navigation conditions.

For defining the CURM, the range of the input space was set as Equation (6).

(6) $$\begin{array}{cc} V=\{\left(v_{l},v_{r}\right)| & v_{l}\in \left[v_{l\_exp}-a_{l}\cdot \Delta t,v_{l\_exp}+a_{l}\cdot \Delta t\right]\\ & v_{r}\in \left[v_{r\_exp}-a_{r}\cdot \Delta t,v_{r\_exp}+a_{r}\cdot \Delta t\right]\} \end{array}$$

(7) $$\begin{array}{c} \mathrm{CURM}\left(v_{l},v_{r}\right)=\left\{v_{l},v_{r}|min\left(Clearance\left(v_{l},v_{r}\right)\right),\frac{{\left(v_{l\_exp}-a_{l}\cdot \Delta t\right)}^{2}}{\sigma_{l}^{2}}+\frac{{\left(v_{r\_exp}-a_{r}\cdot \Delta t\right)}^{2}}{\sigma_{r}^{2}}\leq s\right\} \end{array}$$

In Equation (6), $a_{l}$ and $a_{r}$ are the maximum acceleration values of the left and right wheels, respectively. In order to obtain the value of CURM, Equations (5) and (7) can be used. Algorithm 1 explains how to obtain CURM from Equations (6) and (7). The getUncertainty function of Line 6 returns [$\sigma_{l}$, $\sigma_{r}$].**Algorithm 1:** CURM()1:**for**$v_{l\_ref}=\left(v_{l}-\Delta t\right)$ to $\left(v_{l}+\Delta t\right)$
**do**2: **for**
$v_{r\_ref}=\left(v_{r}-\Delta t\right)$ to $\left(v_{r}+\Delta t\right)$
**do**3:  min = infinite4:  **for**
$v_{l\_ref}=\left(v_{l}-\Delta t\right)$ to $\left(v_{l}+\Delta t\right)$
**do**5:   **for**
$v_{r\_ref}=\left(v_{r}-\Delta t\right)$ to $\left(v_{r}+\Delta t\right)$
**do**6:    [$\sigma_{l}$,$\sigma_{r}$] = getUncertainty ($v_{l\_exp}$,$v_{r\_exp}$,$v_{l\_err}$,$v_{r\_err}$)7:    **if** ($v_{l\_err}^{2}/\sigma_{l}^{2}+v_{r\_err}^{2}/\sigma_{r}^{2}<s$
**and** min > Clearance($v_{l\_ref}$, $v_{r\_ref}$)) **then**8:     min = Clearance($v_{l\_ref}$,$v_{r\_ref}$)9:    **end if**10:   **end for**11:  **end for**12:  CURM [$v_{l\_ref}$,$v_{r\_ref}$] = min13: **end for**14:**end for**

Algorithm 2 shows the A*-based path planner considering the motion uncertainty of mobile robots. The algorithm follows the structure of A*. The OPEN queue is a priority queue, in which the distance traveled is generated. The distances from the start node to the current node are arranged in ascending order. The OPEN queue contains candidate nodes for the trajectory. Certain steps are undertaken before placing the node in the OPEN queue. First, the trajectory is obtained through the motion controller for a candidate node (Line 9). Then, a sampling-based forward simulation is carried out (Line 11). OPEN.Push(node) places the node in the OPEN queue if the collision probability is lower than the threshold K. The CLOSED queue stores nodes, where the child node has been searched for backtracking after arrival at the goal. The rest of the process proceeds as per the basic A* algorithm to obtain an appropriate trajectory.**Algorithm 2:** MakeTrajectoryBasedOnAStar ().1:OPEN.Init()2:CLOSED.Init()3:**if** (isGoal(start) = true) **then**4: return MakeTrajectory(start)5:**end if**6:OPEN.Push(start)7:**while** OPEN.Size() = 0 **do**8: n = OPEN.Pop()9: nodes = Expand(n)10: **for** all the node∈nodes **do**11:  node.trajectory = GenTrajectory(n, node)12:  node.f = n.f + GetLength(node.trajecory) + h(node)13:  collisionProb = SimulationWithMotionUncertainty (node.trajectory)14:  **if** (collisionProb ≥ K) **then**15:   **continue**16:  **end if**17:  **if** (isGoal(node) = true) **then**18:   return MakeTrajectory(node)19:  **end if**20:  **if** (node ∩ CLOSED = ⌀) **then**21:   OPEN.Push(node) with node.f as priority22:  **end if**23: **end for**24: CLOSED.Push(n)25:**end while**

Algorithm 3 corresponds to Line 13 in Algorithm 2. Forward simulations are carried out by considering the uncertainty of robot motion, under the application of the motion controller from the parent node to the candidate child node.**Algorithm 3:** SimulationWithMotionUncertainty ().1:collision = 02:**for** OPEN.Size() = 0 **do**3: [x,y,θ]= Trajectory[0].pose4: **for**
j=1 to Trajectory.Size **do**5:  $[v_{l},v_{r}]=$ Trajectory[j].velocity6:  $\hat{v_{l}}=v_{l}+\frac{1}{2}\sum_{i=1}^{12}rand\left(-\alpha_{1}{v_{l}}^{2},\alpha_{1}{v_{l}}^{2}\right)$7:  $\hat{v_{r}}=v_{r}+\frac{1}{2}\sum_{i=1}^{12}rand\left(-\alpha_{2}{v_{r}}^{2},\alpha_{2}{v_{r}}^{2}\right)$8:  $\hat{v}=\left(v_{l}+v_{r}\right)/2$9:  $\hat{\omega }=\left(v_{r}-v_{l}\right)/b$10:  $x^{\prime }=x-\frac{\hat{v}}{\hat{\omega }}\sin\theta +\frac{\hat{v}}{\hat{\omega }}\sin\left(\theta +\hat{\omega }\Delta t\right)$11:  $y^{\prime }=y-\frac{\hat{v}}{\hat{\omega }}\cos\theta +\frac{\hat{v}}{\hat{\omega }}\cos\left(\theta +\hat{\omega }\Delta t\right)$12:  $\theta^{\prime }=\theta +\hat{\omega }\Delta t$13:  **if** (CollisionCheck(x,y,θ) = true) **then**14:   collision = collision + 115:   **break**16:  **end if**17: **end for**18:**end for**

A velocity motion model [1] is used as the kinematic condition of the two-wheeled mobile robot, as shown in the 6th–12th lines. The trajectory generated in Line 11 of Algorithm 2 is generated by forward simulation using a number of samples. The probability of collision with the obstacle is calculated and returned. The returned value is used to determine whether the node will be placed in the OPEN queue at Line 14 of Algorithm 2.

As the motion uncertainty increases in magnitude, the sample distribution widens. Therefore, the number of colliding samples near the obstacle thus increases. With Algorithms 2 and 3, the paths with a short travel distance are created while safely avoiding obstacles. As a result, a safe and efficient path can be generated by reflecting the uncertainty of the robot motion.

## 4. Simulation and Experimental Results

### 4.1. Measuring the Motion Uncertainty

**Figure 4:**
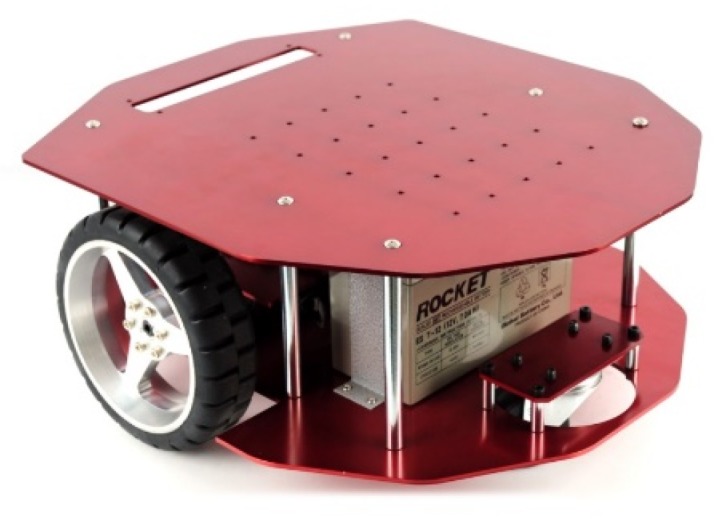
Stella B3.

The measurement range of the motion uncertainty of the mobile robot was based on indoor service robots used in previous studies. Based on the service robots listed in Table 1, the maximum velocity and acceleration were 0.5 m/s and 0.5 m/s${}^{2}$, respectively. The standard deviation of the velocity control error with respect to the velocity and acceleration was obtained. Figure 4 shows the robot used in this experiment. The experiment was repeated with the DWA to measure the velocity and acceleration of approximately 5000 samples.

The method of finding parameters of the motion uncertainty of a two-wheeled mobile robot without actual navigation is as follows. The maximum linear velocity and acceleration of a wheel are determined. A triangular wave that satisfies the maximum linear velocity and acceleration is generated. One of the robot wheels is stationary while the other rotates the robot by the input of a triangular wave. The opposite wheel is also used to obtain the parameters of motion uncertainty in the same way. The motion uncertainty parameter of the robot with the input velocity for each time step and actual measured velocity through the wheel encoder sensor are obtained. When the operating conditions of the robot, such as the motor and controller, are changed, the motion uncertainty parameters are measured again.

**Figure 5:**
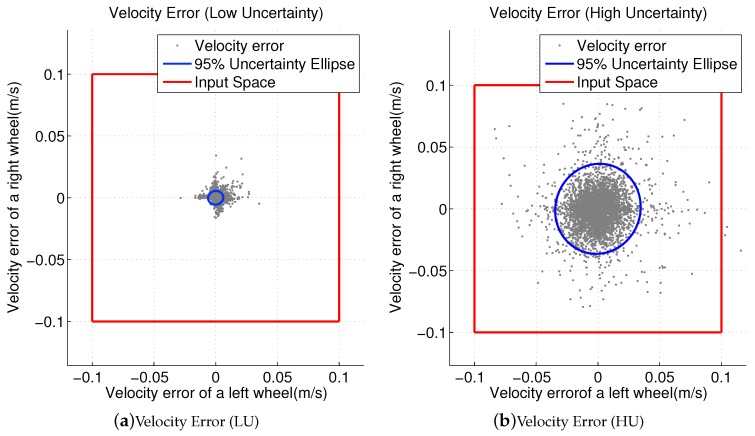
Uncertainty of the experimental resultant velocity in the input space. LU, low uncertainty; HU, high uncertainty.

Table 2 shows the results of motion control error collection for velocity and acceleration. Figure 5 shows the experimental results of the velocity control error measured by encoder sensors. The *x*-axis represents $v_{l\_ref}-v_{l\_exp}$, and the *y*-axis represents $v_{r\_ref}-v_{r\_exp}$. The reference velocity is the wheel velocity as input, and the experimental velocity is the wheel velocity read from the encoder after a control cycle. Figure 5a shows the velocity control error of a low-uncertainty (LU) robot, and Figure 5b shows the velocity control error of a high-uncertainty (HU) robot.

In order to investigate the effect of motion uncertainties, two parameter sets of the wheel velocity controller were tested. The motion uncertainty changes according to different PI gains of the velocity controller. One parameter set showed satisfactory velocity control performance after tuning. This set is called the LU case. The parameters here were [P, I] (current controller gain) = [1, 100] and [P, I] (velocity controller gain) = [0.5, 0.05]. The second parameter set HU demonstrated a less satisfactory control performance. The parameters of this set were [P, I] (current controller gain) = [0.00005, 5.0] and [P, I] (velocity controller gain) = [1.0, 25.0].

From Table 2, it is clear that the control error showed a strong correlation with the desired acceleration. On the other hand, the desired velocities were independent of the experimental control errors. The measured velocity control error was used in Line 7 of Algorithm 1 and Lines 6–7 of Algorithm refalgorithm:makeTrajectory.

### 4.2. CURM and One-Step Simulation

**Figure 6:**
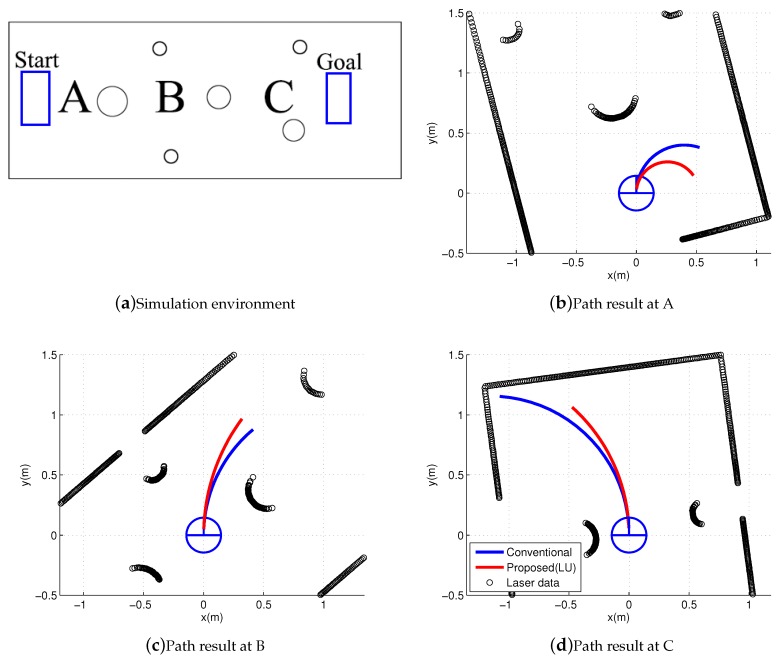
Simulation environment and path results.

This section provides a comparison of the conventional and proposed approaches through simulations. The simulations were performed by applying Algorithm 1. Figure 6a shows a simulation environment. The robot was located at A. Figure 6b–d shows local paths that were generated by the conventional (blue) and proposed (red) schemes. It can be seen that the conventional path was closer to the obstacle than the proposed path. The clearance object of the conventional path was higher than that of the proposed path. However, if the motion uncertainty is taken into account, the conventional path became risky because there was minimal clearance to the lateral direction of the robot.

**Figure 7:**
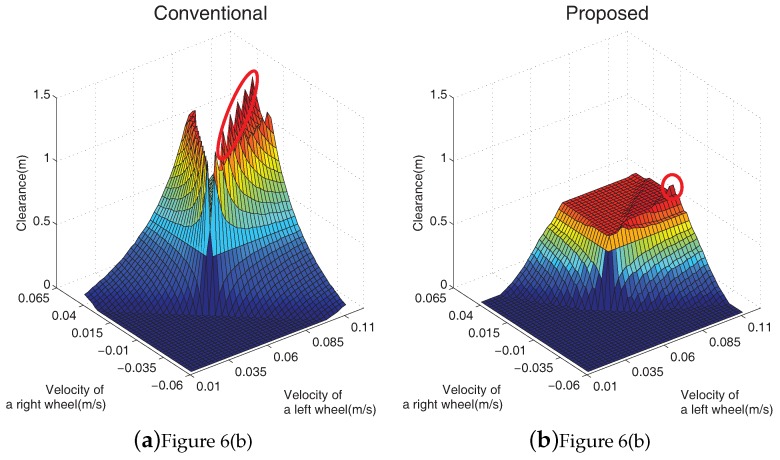

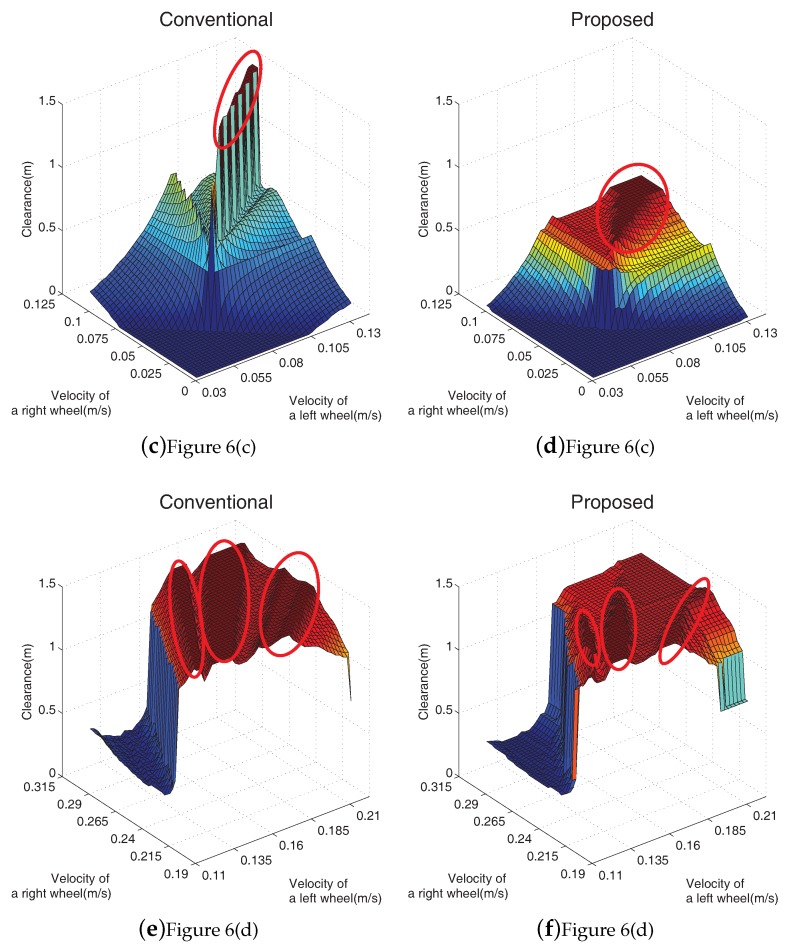
The clearance of the simulation environments.

The CURM from Equation (7) provides the way of obtaining the motion uncertainty during the clearance object computation. The proposed path was generated by the computation of the CURM. The proposed path was safer compared with the conventional path when motion uncertainty existed. However, if the CURM was obtained with the method presented in Figure 7, the expected value of the velocity near the obstacle decreased when the motion uncertainty was considered. Thus, the probability of obstacle collision decreased.

Figure 7 shows the computed clearance object using the conventional scheme and Equation (7). The CURMs at three different locations, A, B, and C of Figure 6, are shown in Figure 7b,d,f, respectively. The red lines in Figure 6b–d are the paths selected by the proposed scheme. Figure 7a,c,e shows the clearance objects without considering the motion uncertainty.

Sharp peaks signify that the robot can be safely driven at the selected input velocities. However, the collision risk dramatically increased if motion uncertainty existed. Dramatic decreases in the clearance objects of Figure 7b,d,f are clearly observed. A decrease in a clearance object implies an increase in the collision risk. Therefore, the computation of CURM was essential in order to guarantee collision-free navigation in practical applications.

### 4.3. Reactive Motion Controller

**Figure 8:**
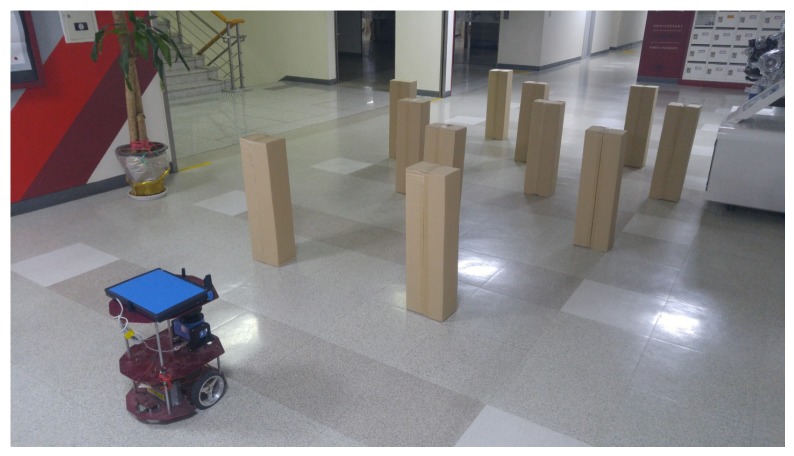
The experimental environment.

Algorithm 1 was experimentally tested. In the cluttered environment of Figure 8, the collision risk of the conventional and proposed methods was investigated. The navigation system was implemented on a Core-Duo 2.53-GHz laptop using the ROS [30] platform and written in C++. The ground truth for the robot pose was obtained by Adaptive Monte-Carlo Localization(AMCL) [31]. For comparison, four indices were used to evaluate the risk of collision: the minimum distance, TTC, minimum distance in the input space, and CRI [14]. The maximum translational velocity of the robot was 0.5 m/s.

**Figure 9:**
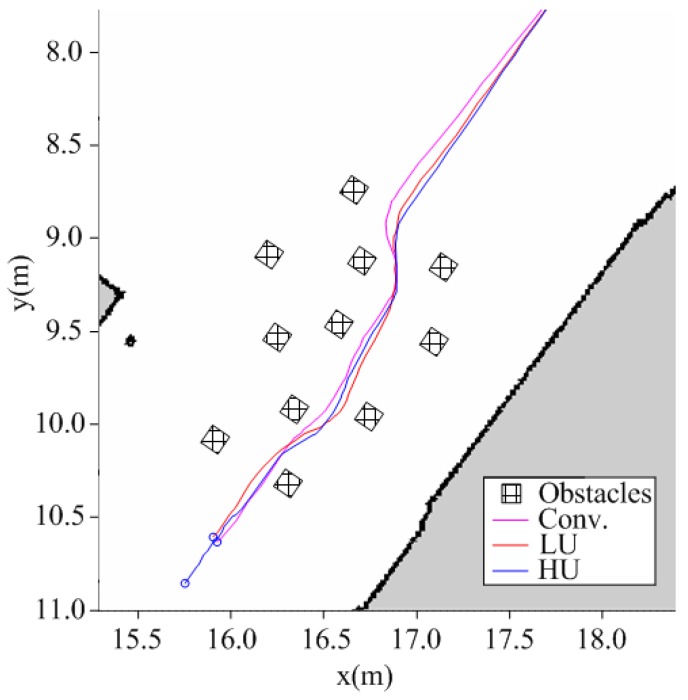
Resultant path.

**Figure 10:**
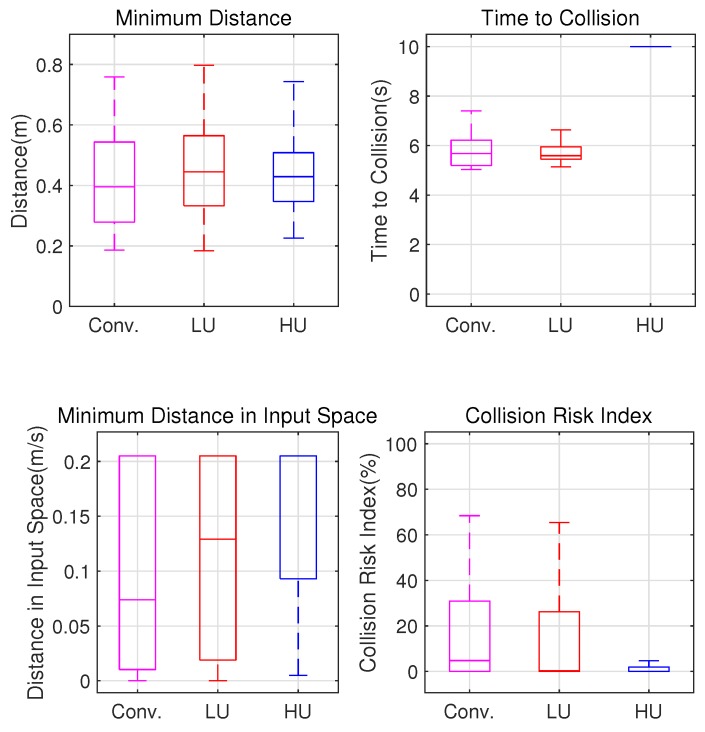
Comparisons of the navigation safety indices.

Figure 9 shows three paths of a robot in a static cluttered environment. Figure 10 compares their navigation safety indices. The conventional path was assumed to be the ideal case, where there was no motion uncertainty. For the LU robot, the safety indices of the proposed method were similar to the conventional method. For the HU robot, the proposed control method was more cautious than the conventional method. This can be concluded from the comparison of the collision risk indices, which indicated the collision risk in the admissible velocity and that the proposed method navigated more safely.

### 4.4. Path Planner

**Figure 11:**
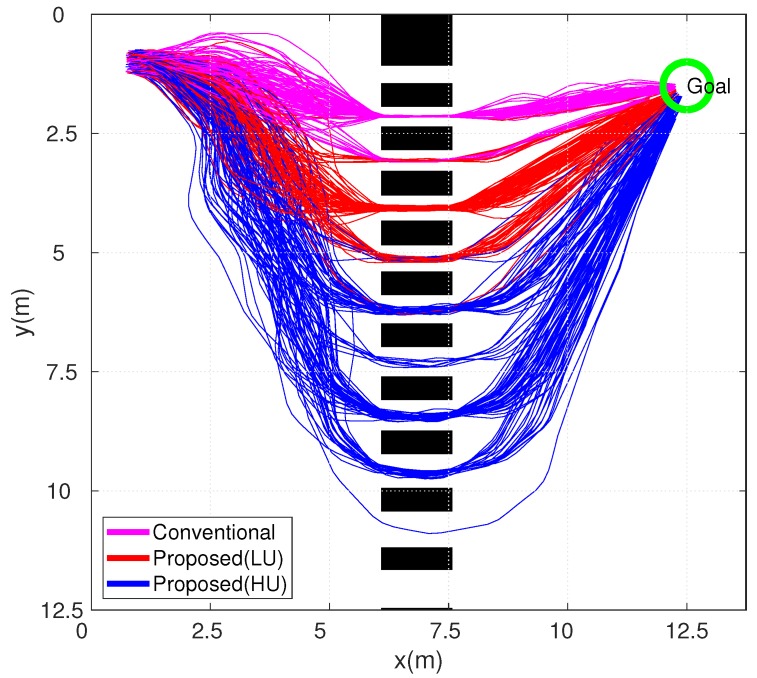
Resultant paths.

Figure 11 shows the simulation results with the proposed path planner in an environment with narrow passages from 0.35–0.85 m. The simulator was developed using the MFClibrary by the authors.

The width of the passage increased when the y-coordinate of the passage increased in Figure 11. The purple lines indicate the resultant paths of the simulation using the conventional method. The red and blue lines indicate the paths of the LU and HU robots, respectively. A total of 100 simulations were carried out for each method. A trajectory can be updated online using the modeled information of the motion uncertainty. In an environment with fixed obstacles, a Core-Duo2 2.53-MHz laptop can generate about one trajectory per second online.

Table 3 shows the quality of paths [32] presented in Figure 11. Using the quality of paths, quantitative comparisons between the conventional and proposed schemes are presented. The collision probability was calculated through the trajectory. The middle and right columns of Table 3 present the approximations that can be computed given the probability distributions along the trajectories. Since the simulation was based on practical motion uncertainty, the conventional method that assumed perfect motion in the collision was excluded.

When the motion uncertainty of the path planner was applied excessively, the quality of paths was 98.7%. When the motion uncertainty of the robot was exactly applied to the path generation, the quality of paths of the LU and HU robots were 97.0% and 89.3%, respectively. When the motion uncertainty of the robot was insufficiently applied, the quality of paths was under 80%. It can be seen that the proposed scheme showed superior performances from the viewpoint of collision safety.

**Figure 12:**
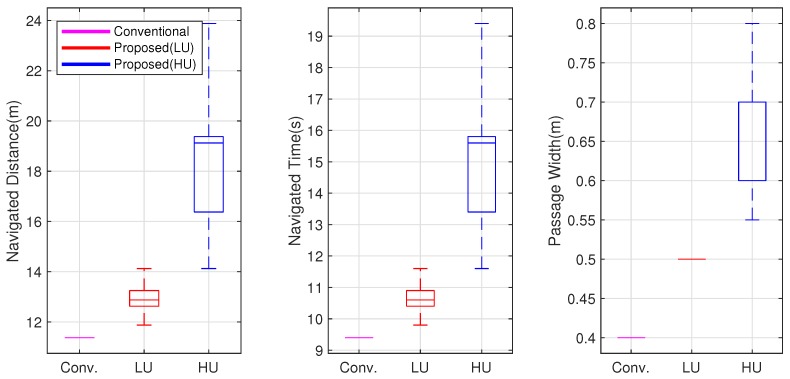
Comparisons of navigated distance, navigated time, and passage width.

Figure 12 shows the distance traveled, time taken, and width of the passage for the 100 simulations. The conventional method tended to generate shorter paths through narrow passages and took up shorter times. The HU robots traveled longer distances and took a longer time. This result signifies that the HU robot moved through wider passages in order to avoid collision under highly uncertain conditions.

**Figure 13:**
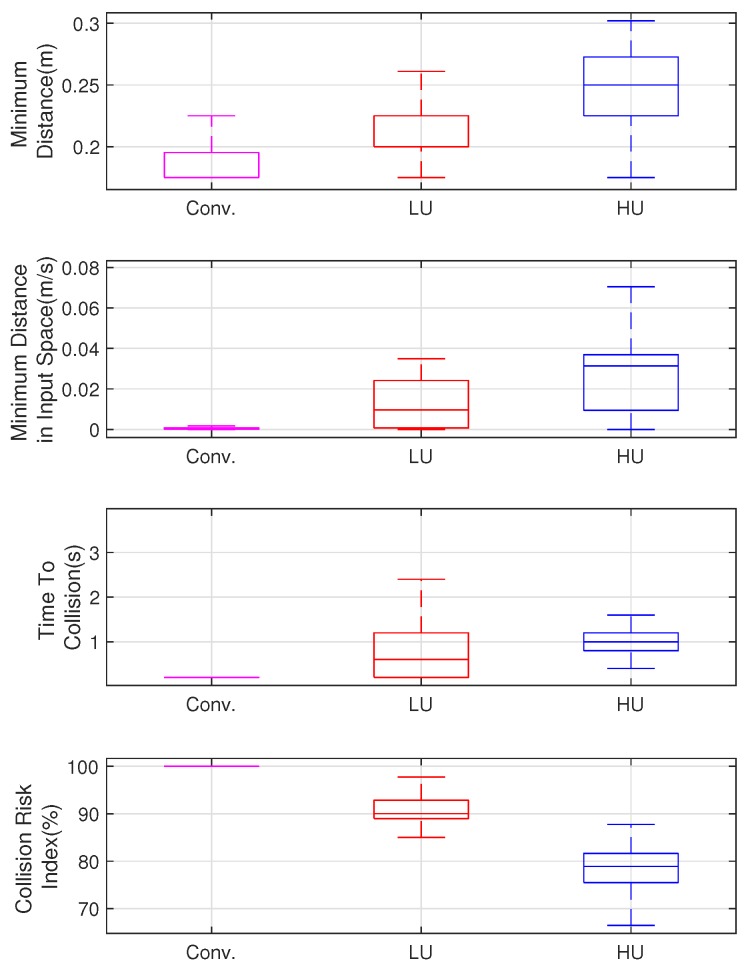
Navigation results: comparison of the safety indices.

Figure 13 shows the simulation results of the safety indices. The results indicate the minimum distance to the obstacles, TTC, the minimum distance from the obstacle in the input space, and CRI when the robot is in a dangerous situation. In all the indices’ results, the conventional approach exhibited the highest collision risk, while the HU robots took the safest paths. This result implies that the consideration of motion uncertainty is extremely significant in real-world applications, where uncertainty is not negligible.

From the simulation results, the motion uncertainty must be explicitly applied to the controller or path planner. It was shown that the safety of the robot improved by considering the motion uncertainty. The application of excessively-extended uncertainty has also demonstrated that the collision risk was reduced. However, the navigation efficiency was reduced. Therefore, it is possible to balance the collision safety and navigation efficiency by accurately applying the motion uncertainty of practical robots.

## 5. Conclusions

In this study, a new motion control scheme toward reliable obstacle avoidance by reflecting the experimental motion uncertainties was proposed. It was shown how the uncertainty of robot motions can be quantitatively modeled based on the performance of the velocity control of a wheel. A controller was proposed where obstacles are extended as much as the motion uncertainty modeled in the input space. The usefulness of the proposed approach was experimentally verified.

The experimental results clearly show that the consideration of the motion uncertainty is essential to successful collision avoidance. A path planner was proposed where the uncertainty of motion is quantitatively reflected. In the environment with multiple narrow passages, the proposed method was compared to the conventional method through generated paths. The conventional method generated shorter paths. However, based on actual motion uncertainty, the conventional method had a high risk of collision in simulation. The path generated by the proposed method may not be the fastest. However, it was generated with both safety and efficiency in consideration. The presented simulation results demonstrated that the proposed method can accurately reflect the motion uncertainty and balance the collision safety with the navigation efficiency of the robot.

## Figures and Tables

**Table 1:** Maximum translational velocities and accelerations of previous service robots.

Year	Robot or Paper	Max. Velocity	Max. Acceleration
1998	Rhino [25]	0.36 m/s	
2000	MINERVA [26]	0.38 m/s	
2004	Jinny [27]	1.0 m/s	0.5 m/s${}^{2}$
2009	Safe Navigation [14]	0.5 m/s	0.8 m/s${}^{2}$
2013	HOSPY [28]	1.0 m/s	
2015	Dual-Tree RRT [29]	1.5 m/s	

**Table 2:** Standard deviation of the control error with respect to the velocity and acceleration.

**Acc. (m/s${}^{2}$)**	**0**	**0.1**	**0.2**	**0.3**	**0.4**	**0.5**
Std. dev. (LU)	0.002	0.005	0.017	0.02	0.031	0.036
Std. dev. (HU)	0.011	0.017	0.074	0.072	0.101	0.109
**Vel. (m/s)**	**0**	**0.1**	**0.2**	**0.3**	**0.4**	**0.5**
Std. dev. (LU)	0.005	0.002	0.006	0.006	0.008	0.003
Std. dev. (HU)	0.018	0.010	0.021	0.02	0.02	0.017

**Table 3:** The quality of paths.

	LU Robot (Avg.)	HU Robot (Avg.)
Conv. Path	72.4%	27.7%
LU Path	97.0%	72.0%
HU Path	98.7%	89.3%
